# Supplemental Nutrition Assistance Program (SNAP)-authorised store marketing environments in Louisiana encourage the selection of less nutritious foods and beverages

**DOI:** 10.1017/jns.2022.60

**Published:** 2022-08-04

**Authors:** Bailey Houghtaling, Melissa Cater, Nila Pradhananga, Denise Holston

**Affiliations:** 1 School of Nutrition and Food Sciences, Louisiana State University (LSU) & LSU Agricultural Center, Baton Rouge, LA 70803, USA; 2 Gretchen Swanson Center for Nutrition, Omaha, NE 68514, USA; 3 Agricultural and Extension Education and Evaluation, Louisiana State University (LSU) & LSU Agricultural Center, Baton Rouge, LA 70803, USA

**Keywords:** Candy, Chips, Food environment, Food marketing, Fruits and vegetables, SNAP, Sugar-sweetened beverages

## Abstract

Marketing influences consumers’ dietary purchases. However, little is known about marketing environments in Supplemental Nutrition Assistance Program (SNAP)-authorised stores. The present study explored SNAP-authorised store marketing environments in Louisiana by rurality, store ownership and store type (*n* 42). Sampling methods were designed to include randomly selected stores in each geographic area of the state. The *GroPromo* was used to measure placement, promotion, and child-focused aspects of marketing strategies used for healthier (fruits and vegetables) and less healthy products (chips, candy, sugar-sweetened beverages, child-focused cereal) in medium- and high-prominence marketing areas. In using multivariate analysis of variance (MANOVA) (*P* < 0⋅05) for data analysis, variations in *GroPromo* scores were found among SNAP-authorised stores by rurality (*P* < 0⋅05) and store ownership (*P* < 0⋅001); no differences were found by store type (*P* > 0⋅05). Future research, practice and policy strategies are required to understand the influence of marketing environments on SNAP participants’ dietary quality and to design responsive public health interventions.

## Introduction

Aspects of marketing environments, such as the placement and promotion of healthier or less healthy foods and beverages (i.e. based on the 2015–2020 Dietary Guidelines for Americans^(1)^) influence consumers’ dietary purchasing decisions^(2,3)^. However, little is currently known about marketing environments in Supplemental Nutrition Assistance Program (SNAP)-authorised stores. The United States Department of Agriculture (USDA) SNAP serves populations with lower income by providing supplemental income for food and beverage purchases at authorised stores^(4)^. The SNAP also has a wide reach to US children; around 7⋅4 billion households with children participated in the SNAP in 2019^(4)^.

The USDA is a central supporter of nutrition security efforts in the United States (US), defined as, ‘consistent access, availability, and affordability of foods and beverages that promote well-being and prevent (and if needed, treat) disease’^(5,6)^. Equity is a core aspect of achieving nutrition security^(5)^, and while food and beverage marketing is not explicitly addressed, consumers with lower income and those living in predominantly ethnic and racial minority communities seem disproportionately targeted with marketing strategies for products higher in saturated fats, added sugars and sodium^(7–9)^. Children, who are key influencers of household dietary purchases^(10)^, are also prime targets for less healthy food and beverage product marketing^(9,11)^.

These factors could contribute to low diet quality scores documented among SNAP populations^(2,12)^. However, researchers using food environment assessment tools to examine SNAP-authorised stores have largely focused on healthy *v*. less healthy product availability, affordability and quality^(13–15)^. The present study builds upon this work through an exploration of placement, promotion and child-focused marketing strategies used to sell an assortment of healthier and less healthy foods and beverages among SNAP-authorised stores in Louisiana. A research-to-practice approach was used to inform healthy food retail strategies in the state.

## Methods

The present study used a cross-sectional quantitative descriptive design. In-store assessments were used to capture properties of marketing environments among stores authorised to accept SNAP benefits in Louisiana. Study details are described below in brief. For a more comprehensive account, see Houghtaling *et al.*^(13)^.

### Setting

An estimated 26 % of children under the age of 18 live in poverty in Louisiana, which is well above the US average (17 %)^(16)^. Louisiana Cooperative Extension Services administers SNAP-Education, the educational component of the SNAP, and is a pioneering partnership and programming efforts to improve the accessibility of healthy options in Louisiana food retail settings^(17)^. The present study was intended to inform these efforts.

### Sampling

A public database of SNAP-authorised store name and location data was used for sampling^(18)^. Five geographic regions of the state were prioritised (i.e. southwest, southeast, central, northeast and northwest Louisiana) that align with Cooperative Extension Services programme areas. Using the USDA 2013 Rural-Urban Continuum Code (RUCC), the most urban (RUCC 1) and most rural parishes (RUCC 8 and 9) were selected^(19)^. Using a random number generator, a rural parish classified as either RUCC 6 or 7 in each region was also selected. Then, based on a power calculation, five SNAP-authorised stores were randomly selected in each chosen parish (with one required to be a grocery store) for a planned sample of 75 SNAP-authorised stores^(13)^.

### Measure

The *GroPromo*^(20)^ paper survey was implemented in each store with permission between October 2019 and March 2020, at which point data collection ceased due to the COVID-19 pandemic. This tool was examined for inter-rater reliability and construct validity in a sample of US grocery stores^(20)^ and was therefore selected for use in this study. The *GroPromo* documents placement and promotion strategies used to sell chips, candy, soda, child-focused cereal and fruits and vegetables (FVs) in several in-store locations.

Because this tool was used alongside other food environment assessments^(13)^, small changes were made to reduce implementor burden and the time spent in sampled stores. Lower-prominence marketing locations, per the *GroPromo*, were excluded from data collection: outside the store entrance; main store aisles and the inside store perimeter^(20)^. Medium- and high-prominence locations were prioritised: the store entrance(s) (immediate 10 foot area); store endcaps (facing towards and away from checkout areas); islands (i.e. movable displays) and store checkouts (sides and endcaps)^(20)^.

Small adaptations to the food and beverage categories were also made to improve the tool relevance for public health nutrition. In addition to fresh FV, this category was expanded to include canned products in 100 % juice or without sauces/seasonings. The soda category was expanded to include any sugar-sweetened beverage (SSB), defined as non-alcoholic beverages with added sugars^(8)^. At each of the selected store locations, trained researchers identified displayed products and documented several promotional features as measured by *GroPromo*: size or whether the display was larger than normal for the store; themed promotions, such as for holidays or sporting events; if there were more than five varieties of the food or beverage item in a display; whether the display was in reach of children (<4 feet from the ground); and any child-focused marketing features such as cartoon characters or prizes, for example^(20)^.

A scoring scheme was developed by the study team in order to classify and compare marketing attributes among healthier and less healthy foods and beverages in SNAP-authorised stores, such that stores would score higher (or more favourably) if several varieties of FVs were placed in a greater number of store marketing locations and accompanied by larger sized, themed displays, that were both within reach of and focused towards children. Thus, SNAP-authorised marketing environments would score lower (or less favourably) with a greater number and variety of candies, chips, SSB and child-focused cereals in larger, themed displays placed near to and focused towards children. Scores across *GroPromo* categories placement (range −288 to 288), promotion (range −360 to 360) and child-focused marketing (range −240 to 240) are summed to reflect total scores (range −888 to 888). These scoring details are available in the Supplementary material.

### Data analysis

Data were analysed using IBM SPSS Statistics version 27 (IBM Corp, Armon, NY, USA). Descriptive statistics including averages and standard deviations were calculated. In accordance with the prior study^(13)^, and given a lack of information about SNAP-authorised store marketing environments in general, multivariate analysis of variance (MANOVA) was used to explore if there were differences in total *GroPromo* scores or placement, promotion and child marketing subscores between: urban (defined as RUCC 1–3^(19)^) and rural (defined as RUCC 4–9^(19)^) SNAP-authorised stores; independently owned and corporate/chain-owned SNAP-authorised stores; and different types of stores (i.e. grocery, dollar and convenience). Pillai's trace was used for the omnibus multivariate test of significance (*a priori*, *P* < 0⋅05). Next, univariate tests of between-subject effects were used to find specific points of difference among groups.

## Results and discussion

The abbreviated *GroPromo* was implemented in 42 SNAP-authorised stores, including grocers (*n* 12), convenience stores (*n* 17), dollar stores (*n* 11), a drug store (*n* 1) and a butcher (*n* 1). Implementation took on average 13 (sd ± 11) minutes. Most of the SNAP-authorised stores were rural (*n* 29; 72⋅5 %) and corporate/chain-owned (*n* 22; 52 %). The average number of displays documented in medium- and high-prominence marketing locations for candy, chips, SSB, child-focused cereal and FVs are shown in Table 1. FVs were, on average, rarely placed in medium- or high-prominence locations in SNAP-authorised stores (Table 1). This finding aligns with Kerr *et al.* regarding less healthier products more often placed in prominent marketing locations relative to FVs^(20)^. SSB were prominent throughout store locations, aside from checkout sides where candies were most often documented (Table 1).

**Table 1.** Displays for healthier and less healthy food and beverage products documented in medium- and high-prominence marketing locations in Supplemental Nutrition Assistance Program (SNAP)-authorised stores in Louisiana, USA^a^

Product	Marketing location											
	Entrance(s)		Endcaps facing checkouts		Endcaps facing away from checkouts		Islands		Checkout endcaps		Checkout sides	
	M sd	Range	M sd	Range	M sd	Range	M sd	Range	M sd	Range	M sd	Range
Candy	0⋅3 ± 0⋅8	0–3	0⋅5 ± 0⋅8	0–3	0⋅1 ± 0⋅5	0–3	0⋅7 ± 1⋅1	0–4	0⋅4 ± 0⋅8	0–3	2⋅1 ± 2⋅2	0–10
Chips	0⋅6 ± 1⋅6	0–7	0⋅9 ± 1	0–4	0⋅6 ± 0⋅8	0–4	1⋅4 ± 2⋅2	0–10	0⋅5 ± 0⋅8	0–10	0⋅8 ± 1⋅3	0–6
SSB^b^	1⋅1 ± 1⋅5	0–8	1⋅1 ± 1⋅2	0–3	1⋅6 ± 1⋅6	0–8	2 ± 2⋅9	0–14	1⋅1 ± 2	0–7	1 ± 1⋅7	0–6
CFC^c^	0⋅02 ± 0⋅2	0–1	0⋅1 ± 0⋅3	0–1	0⋅3 ± 0⋅5	0–2	0⋅1 ± 0⋅6	0–3	0	–	0	−
FVs^d^	0	0	0⋅02 ± 0⋅2	0–1	0⋅1 ± 0⋅3	0–2	0⋅1 ± 0⋅5	0–2	0	–	0⋅02 ± 0⋅2	0–1

a Means (M) and standard deviations (sd).

b Sugar-sweetened beverages, defined as non-alcoholic drinks with added sugars.

c Child-focused cereal (e.g. with cartoons or caricatures targeted towards children).

d Fruits and vegetables.

Total *GroPromo* scores ranged from 6 to 127 in this research; placement subscores ranged from −65 to 62; promotion subscores ranged from 18 to 105; and child marketing subscores ranged from −26 to 40. Pillai's trace indicated a statistically significant effect of rurality (*V* = 0⋅3, *F*(3,28) = 4⋅0, *P* = 0⋅02). Total *GroPromo* scores or child marketing subscores did not differ between urban and rural SNAP-authorised stores. However, rural SNAP-authorised stores scored lower (less favourably) than urban stores on the placement subscore (−5⋅6 ± 32⋅4 *v*. 8⋅6 ± 37⋅8, respectively; *P* = 0⋅004). Alternatively, urban SNAP-authorised stores scored lower (less favourably) than rural stores on the promotion subscore (41⋅3 ± 26⋅8 *v*. 55⋅1 ± 20⋅5, respectively; *P* = 0⋅01) (see Table 2).

**Table 2.** Differences in *GroPromo* total scores and placement, promotion and child marketing subscores among Supplemental Nutrition Assistance Program (SNAP)-authorised stores in Louisiana (*n* 40^a^)

Categories	Total score		Placement subscore		Promotion subscore		Child marketing subscore	
	M	sd	M	sd	M	sd	M	sd
All SNAP-authorised stores		47⋅3	±26⋅5	−1⋅7	±34⋅1	51⋅3	±22⋅9	−2⋅4	±13⋅2
Location	Urban	46⋅0	±29⋅3	8⋅6^b^	±37⋅8	41⋅3^c^	±26⋅8	−3⋅9	±10⋅7
	Rural	47⋅8	±26⋅0	−5⋅6	±32⋅4	55⋅1	±20⋅5	−1⋅8	±14⋅1
SNAP-authorised store ownership	Corporate/chain-owned	31⋅4^d^	±12⋅1	−25⋅2^e^	±19⋅9	60⋅8^f^	±22⋅4	−4⋅2	±14⋅0
	Independently owned	68⋅8	±25⋅7	30⋅2	±20⋅3	38⋅4	±16⋅7	0⋅2	±11⋅8
SNAP-authorised store type	Grocery	41⋅6	±31⋅4	−18⋅6	±32⋅1	62⋅7	±21⋅9	−2⋅5	±17⋅2
	Dollar	33⋅1	±13⋅8	−29⋅2	±10⋅6	65⋅5	±17⋅9	−3⋅3	±17⋅6
	Convenience	60⋅5	±23⋅9	28⋅1	±19⋅4	34⋅0	±13⋅9	−1⋅6	±4⋅9

a SNAP-authorised drug and butcher stores were excluded from the analysis due to the low sample size.

b Test of between-subject effects (*F* = 9⋅9, *P* = 0⋅004).

c Test of between-subject effects (*F* = 7⋅5, *P* = 0⋅01).

d Test of between-subject effects (*F* = 19⋅3, *P* < 0⋅001).

e Test of between-subject effects (*F* = 62⋅1, *P* < 0⋅001).

f Test of between-subject effects (*F* = 5⋅4, *P* = 0⋅027).

Based on how the *GroPromo* was scored, these findings indicate that, on average, and compared to urban stores, rural SNAP-authorised stores had a greater number of less healthy displays in medium- and high-prominence locations (i.e. candy, chips, SSB, child-focused cereals) with high product variety and fewer FVs. Urban SNAP-authorised stores had a greater number of large, branded and themed (i.e. sports, holiday) displays for less healthy products than rural stores (Table 2). These differences caused similar total scores, although show interesting variations that require more investigation in future research, i.e. potentially resulting from store size and location, differences in manufacturer distribution/contracts, or strategy for consumer sales in lower or higher populated areas.

Pillai's trace also indicated a statistically significant effect of the business model (*V* = 0⋅7, *F*(3,28) = 4⋅0, *P* < 0⋅001) on *GroPromo* scores; total scores and placement and promotion subscores were found to differ (Table 2). Child marketing scores did not statistically differ by the SNAP-authorised ownership model. Corporate/chain-owned SNAP-authorised stores, on average, scored lower (less favourably) than independently owned stores overall (total *GroPromo* score) (31⋅4 ± 12⋅1 *v*. 68⋅8 ± 25⋅7, respectively; *P* < 0⋅001) and on the placement subscore (−25⋅2 ± 19⋅9 *v*. 30⋅2 ± 20⋅3, respectively; *P* < 0⋅001). Independent SNAP-authorised stores scored, on average, lower (less favourably) than corporate/chain-owned stores on the promotion subscore (38⋅4 ± 60⋅8 *v*. 60⋅8 ± 22⋅4, respectively; *P* = 0⋅027). Subscore differences indicate corporate/chain SNAP-authorised stores had a greater number of less healthy displays with high product variety, while independent stores had larger, branded and themed displays for less healthy products in comparison.

In prior research that examined the availability, price and quality of healthier *v.* less healthy foods and beverages among this sample, independently owned SNAP-authorised stores scored worse than corporate/chain-stores^(13)^. Given the results of this research, holistic store assessments are recommended for characterising healthy and less healthy properties of SNAP-authorised stores, as more salient marketing environments in corporate/chain-owned stores may negatively influence the dietary quality of consumers’ purchases, for example, even though healthier products may be more available, affordable and higher quality than items found in independently owned stores. This rationale can also be used to interpret the finding that no differences in total *GroPomo* scores or subscores were found by store type. For example, grocery stores typically are considered ‘healthier’ than other formats yet are found in this research to have just as poor marketing environments as dollar and convenience stores.

Overall, SNAP-authorised marketing environments scored poorly overall, and show that child-focused marketing strategies for less healthy products are pervasive throughout urban and rural, corporate/chain- and independently owned, and grocery, dollar, and convenience SNAP-authorised stores in Louisiana. This aligns with work finding child-focused marketing for less healthy products has become more pronounced over time despite industry pledges to improve^(11)^. These factors and their relation to SNAP consumer food and beverage purchasing require more investigation using standardised metrics^(3)^. This research can serve as a model for future work, as to our knowledge no other study has implemented the *GroPromo* beyond the initial validation study^(20)^, and strategies to monitor food systems are required to assess progress towards meeting public health nutrition goals^(3)^. More data is needed from diverse geographic locations and among a larger variety of stores. Qualitative inquiry among SNAP-authorised retailers is also recommended to understand the drivers of marketing environments in diverse contexts and to understand how healthy food retail strategies might be used to improve the overall marketing environment to include healthier options in prominent store locations.

In addition, other SNAP-ed implementing agencies may find this approach useful for the needs assessment process, which is required by the funding agency, and can inform healthy food retail strategies. The *GroPromo* was time-efficient and relatively easy to administer. Cooperative Extension Services Agents can use this tool to quickly assess store marketing environments to inform programming efforts, which will build on store assets or challenges in partnership with SNAP-authorised retailers^(17)^. This tool could also be amended for future research and practice efforts depending on community needs. For example, water could be used to expand the healthier products assessed by the *GroPromo* beyond FVs. Alcohol could also be added, given alcoholic beverages were observed frequently in branded or themed displays.

While the results of this research add to a scant literature base on SNAP-authorised marketing environments, there are several limitations to consider. The *GroPomo* was implemented cross-sectionally, based on researcher availability to travel to a store location and/or depending on whether an owner/manager was onsite to provide permission (stores were visited up to three times in this instance^(13)^). Therefore, implementing store assessments at a standard time per month was not possible; a limitation because SSB marketing strategies, in particular, have been found to vary throughout the month based on SNAP benefit issuance periods^(7)^. The *GroPomo* should be used in future research to understand this phenomenon among a variety of less healthy products. Also, the full *GroPomo* was not used, as low-prominence locations were omitted from the data collection plan to save time. However, Kerr *et al.* found few less healthy or healthier products in low-prominence locations relative to medium- or high-prominence locations^(20)^, and indicate results of this research were unlikely to change if the full tool had been used.

Stores not authorised to accept SNAP were not a focus in this research. SNAP-authorisation standards are fairly limited, and most community food retail settings tend to be SNAP-authorised. However, there could be important differences worthy of investigation in future work. Last, the scoring protocol was helpful to assess differences between a variety of SNAP-authorised store contexts, which is important for tailored public health solutions. However, the theoretical range of scores does not reflect reality. For example, the top *GroPromo* score would require large displays of a variety of FVs in each prominent marketing location, accompanied by branding, themes and child-focused marketing strategies, without the presence of any other less healthy item. This is clearly an unrealistic goal. To further refine the scoring scheme, by determining what a ‘healthy’ marketing score is, both overall and among subscores, the *GroPromo* will need to be implemented in more settings for comparison.

In conclusion, more research using the *GroPromo* is warranted to characterise and compare attributes of SNAP-authorised marketing environments. Given USDA's stated focus on nutrition security^(5,6)^, improvements to SNAP-authorised marketing environments, especially in socially and economically disadvantaged community settings, could prove critical for fully realising nutrition security goals.

## Supplementary material

For supplementary material accompanying this paper visit https://doi.org/10.1017/jns.2022.60. click here to view supplementary material
