# Supplementary material for: Supplemental Nutrition Assistance Program (SNAP)-authorised store marketing environments in Louisiana encourage the selection of less nutritious foods and beverages
Source: J Nutr Sci. 2022 Aug 4;11:e64. doi: 10.1017/jns.2022.60 (PMC9379931; doi:10.1017/jns.2022.60)
Supplement: Supplementary file 1 [file S204867902200060Xsup001.docx]

| **1. PLACEMENT SCORING** | | | | | | | |
| --- | --- | --- | --- | --- | --- | --- | --- |
| Location | Product | Points | Variety (*5+ items*) | Points or N/A* | Healthy Alternative (*diet/low-fat/low sugar*) | Points or N/A* | Row Total |
| **Entrance** | 0 CA = 4  1-3 CA = (-1)  4-6 CA = (-2)  7-9 CA = (-3)  ≥10 CA = (-4) | Range -4 to 4 | 0% CA = 4  1-25% CA = (-1)  26-50% CA = (-2)  51-75% CA = (-3)  76-100% CA = (-4) | Range -4 to 4 | 0% CA = (-4)  1-25% CA = 1  26-50% CA = 2  51-75% CA = 3  76-100% CA = 4 | Range -4 to 4 | Range -12 to 12 |
|  | 0 CH = 4  1-3 CH = (-1)  4-6 CH = (-2)  7-9 CH = (-3)  ≥10 CH = (-4) | Range -4 to 4 | 0% CH = 4  1-25% CH = (-1)  26-50% CH = (-2)  51-75% CH = (-3)  76-100% CH = (-4) | Range -4 to 4 | 0% CH = (-4)  1-25% CH = 1  26-50% CH = 2  51-75% CH = 3  76-100% CH = 4 | Range -4 to 4 | Range -12 to 12 |
|  | 0 SSB = 4  1-3 SSB = (-1)  4-6 SSB = (-2)  7-9 SSB = (-3)  ≥10 SSB = (-4) | Range -4 to 4 | 0% SSB = 4  1-25% SSB = (-1)  26-50% SSB = (-2)  51-75% SSB = (-3)  76-100% SSB = (-4) | Range -4 to 4 | 0% SSB = (-4)  1-25% SSB = 1  26-50% SSB = 2  51-75% SSB = 3  76-100% SSB = 4 | Range -4 to 4 | Range -12 to 12 |
|  | 0 CFC = 4  1-3 CFC = (-1)  4-6 CFC = (-2)  7-9 CFC = (-3)  ≥10 CFC = (-4) | Range -4 to 4 | 0% CFC = 4  1-25% CFC = (-1)  26-50% CFC = (-2)  51-75% CFC = (-3)  76-100% CFC = (-4) | Range -4 to 4 | 0% CFC = (-4)  1-25% CFC = 1  26-50% CFC = 2  51-75% CFC = 3  76-100% CFC = 4 | Range -4 to 4 | Range -12 to 12 |
|  | 0 FV = (-4)  1-3 FV = (-3)  4-6 FV = (-2)  7-9 FV = (-1)  ≥10 FV = 4 | Range -4 to 4 | 0% FV = (-4)  1-25% FV = (1)  26%-50% FV = (2)  51-75% FV = (3)  76-100% FV = (4) | Range -4 to 4 | N/A |  |  |
| **ENTRANCE PLACEMENT SCORE** | | | | | | | **Range -48 to 48** |
| **End Cap A** | 0 CA = 4  1-3 CA = (-1)  4-6 CA = (-2)  7-9 CA = (-3)  ≥10 CA = (-4) | Range -4 to 4 | 0% CA = 4  1-25% CA = (-1)  26-50% CA = (-2)  51-75% CA = (-3)  76-100% CA = (-4) | Range -4 to 4 | 0% CA = 4  1-25% CA = (-1)  26-50% CA = (-2)  51-75% CA = (-3)  76-100% CA = (-4) | Range -4 to 4 | Range -12 to 12 |
|  | 0 CH = 4  1-3 CH = (-1)  4-6 CH = (-2)  7-9 CH = (-3)  ≥10 CH = (-4) | Range -4 to 4 | 0% CH = 4  1-25% CH = (-1)  26-50% CH = (-2)  51-75% CH = (-3)  76-100% CH = (-4) | Range -4 to 4 | 0% CH = 4  1-25% CH = (-1)  26-50% CH = (-2)  51-75% CH = (-3)  76-100% CH = (-4) | Range -4 to 4 | Range -12 to 12 |
|  | 0 SSB = 4  1-3 SSB = (-1)  4-6 SSB = (-2)  7-9 SSB = (-3)  ≥10 SSB = (-4) |  | 0% SSB = 4  1-25% SSB = (-1)  26-50% SSB = (-2)  51-75% SSB = (-3)  76-100% SSB = (-4) |  | 0% SSB = 4  1-25% SSB = (-1)  26-50% SSB = (-2)  51-75% SSB = (-3)  76-100% SSB = (-4) | Range -4 to 4 | Range -12 to 12 |
|  | 0 CFC = 4  1-3 CFC = (-1)  4-6 CFC = (-2)  7-9 CFC = (-3)  ≥10 CFC = (-4) | Range -4 to 4 | 0% CFC = 4  1-25% CFC = (-1)  26-50% CFC = (-2)  51-75% CFC = (-3)  76-100% CFC = (-4) | Range -4 to 4 | 0% CFC = 4  1-25% CFC = (-1)  26-50% CFC = (-2)  51-75% CFC = (-3)  76-100% CFC = (-4) | Range -4 to 4 | Range -12 to 12 |
|  | 0 FV = (-4)  1-3 FV = (-3)  4-6 FV = (-2)  7-9 FV = (-1)  ≥10 FV = 4 | Range -4 to 4 | 0% FV = (-4)  1-25% FV = 1  26%-50% FV = 2  51-75% FV = 3  76-100% FV = 4 | Range -4 to 4 | N/A |  |  |
| **END CAP A PLACEMENT SCORE** | | | | | | | Range -48 to 48 |
| **End Cap B** | 0 CA = 4  1-3 CA = (-1)  4-6 CA = (-2)  7-9 CA = (-3)  ≥10 CA = (-4) | Range -4 to 4 | 0% CA = 4  1-25% CA = (-1)  26-50% CA = (-2)  51-75% CA = (-3)  76-100% CA = (-4) | Range -4 to 4 | 0% CA = 4  1-25% CA = (-1)  26-50% CA = (-2)  51-75% CA = (-3)  76-100% CA = (-4) | Range -4 to 4 | Range -12 to 12 |
|  | 0 CH = 4  1-3 CH = (-1)  4-6 CH = (-2)  7-9 CH = (-3)  ≥10 CH = (-4) | Range -4 to 4 | 0% CH = 4  1-25% CH = (-1)  26-50% CH = (-2)  51-75% CH = (-3)  76-100% CH = (-4) | Range -4 to 4 | 0% CH = 4  1-25% CH = (-1)  26-50% CH = (-2)  51-75% CH = (-3)  76-100% CH = (-4) | Range -4 to 4 | Range -12 to 12 |
|  | 0 SSB = 4  1-3 SSB = (-1)  4-6 SSB = (-2)  7-9 SSB = (-3)  ≥10 SSB = (-4) | Range -4 to 4 | 0% SSB = 4  1-25% SSB = (-1)  26-50% SSB = (-2)  51-75% SSB = (-3)  76-100% SSB = (-4) | Range -4 to 4 | 0% SSB = 4  1-25% SSB = (-1)  26-50% SSB = (-2)  51-75% SSB = (-3)  76-100% SSB = (-4) | Range -4 to 4 | Range -12 to 12 |
|  | 0 CFC = 4  1-3 CFC = (-1)  4-6 CFC = (-2)  7-9 CFC = (-3)  ≥10 CFC = (-4) | Range -4 to 4 | 0% CFC = 4  1-25% CFC = (-1)  26-50% CFC = (-2)  51-75% CFC = (-3)  76-100% CFC = (-4) | Range -4 to 4 | 0% CFC = 4  1-25% CFC = (-1)  26-50% CFC = (-2)  51-75% CFC = (-3)  76-100% CFC = (-4) | Range -4 to 4 | Range -12 to 12 |
|  | 0 FV = (-4)  1-3 FV = (-3)  4-6 FV = (-2)  7-9 FV = (-1)  ≥10 FV = 4 | Range -4 to 4 | 0% FV = (-4)  1-25% FV = 1  26%-50% FV = 2  51-75% FV = 3  76-100% FV = 4 | Range -4 to 4 | N/A |  |  |
| **END CAP B PLACEMENT SCORE** | | | | | | | **Range -48 to 48** |
| **Island** | 0 CA = 4  1-3 CA = (-1)  4-6 CA = (-2)  7-9 CA = (-3)  ≥10 CA = (-4) | Range -4 to 4 | 0% CA = 4  1-25% CA = (-1)  26-50% CA = (-2)  51-75% CA = (-3)  76-100% CA = (-4) | Range -4 to 4 | 0% CA = 4  1-25% CA = (-1)  26-50% CA = (-2)  51-75% CA = (-3)  76-100% CA = (-4) | Range -4 to 4 | Range -12 to 12 |
|  | 0 CH = 4  1-3 CH = (-1)  4-6 CH = (-2)  7-9 CH = (-3)  ≥10 CH = (-4) | Range -4 to 4 | 0% CH = 4  1-25% CH = (-1)  26-50% CH = (-2)  51-75% CH = (-3)  76-100% CH = (-4) | Range -4 to 4 | 0% CH = 4  1-25% CH = (-1)  26-50% CH = (-2)  51-75% CH = (-3)  76-100% CH = (-4) | Range -4 to 4 | Range -12 to 12 |
|  | 0 SSB = 4  1-3 SSB = (-1)  4-6 SSB = (-2)  7-9 SSB = (-3)  ≥10 SSB = (-4) | Range -4 to 4 | 0% SSB = 4  1-25% SSB = (-1)  26-50% SSB = (-2)  51-75% SSB = (-3)  76-100% SSB = (-4) | Range -4 to 4 | 0% SSB = 4  1-25% SSB = (-1)  26-50% SSB = (-2)  51-75% SSB = (-3)  76-100% SSB = (-4) | Range -4 to 4 | Range -12 to 12 |
|  | 0 CFC = 4  1-3 CFC = (-1)  4-6 CFC = (-2)  7-9 CFC = (-3)  ≥10 CFC = (-4) | Range -4 to 4 | 0% CFC = 4  1-25% CFC = (-1)  26-50% CFC = (-2)  51-75% CFC = (-3)  76-100% CFC = (-4) | Range -4 to 4 | 0% CFC = 4  1-25% CFC = (-1)  26-50% CFC = (-2)  51-75% CFC = (-3)  76-100% CFC = (-4) | Range -4 to 4 | Range -12 to 12 |
|  | 0 FV = (-4)  1-3 FV = (-3)  4-6 FV = (-2)  7-9 FV = (-1)  ≥10 FV = 4 | Range -4 to 4 | 0% FV = (-4)  1-25% FV = 1  26%-50% FV = 2  51-75% FV = 3  76-100% FV = 4 | Range -4 to 4 | N/A |  |  |
| **ISLAND PLACEMENT SCORE** | | | | | | | **Range -48 to 48** |
| **Checkout End** | 0 CA = 4  1-3 CA = (-1)  4-6 CA = (-2)  7-9 CA = (-3)  ≥10 CA = (-4) | Range -4 to 4 | 0% CA = 4  1-25% CA = (-1)  26-50% CA = (-2)  51-75% CA = (-3)  76-100% CA = (-4) | Range -4 to 4 | 0% CA = 4  1-25% CA = (-1)  26-50% CA = (-2)  51-75% CA = (-3)  76-100% CA = (-4) | Range -4 to 4 | Range -12 to 12 |
|  | 0 CH = 4  1-3 CH = (-1)  4-6 CH = (-2)  7-9 CH = (-3)  ≥10 CH = (-4) | Range -4 to 4 | 0% CH = 4  1-25% CH = (-1)  26-50% CH = (-2)  51-75% CH = (-3)  76-100% CH = (-4) | Range -4 to 4 | 0% CH = 4  1-25% CH = (-1)  26-50% CH = (-2)  51-75% CH = (-3)  76-100% CH = (-4) | Range -4 to 4 | Range -12 to 12 |
|  | 0 SSB = 4  1-3 SSB = (-1)  4-6 SSB = (-2)  7-9 SSB = (-3)  ≥10 SSB = (-4) | Range -4 to 4 | 0% SSB = 4  1-25% SSB = (-1)  26-50% SSB = (-2)  51-75% SSB = (-3)  76-100% SSB = (-4) | Range -4 to 4 | 0% SSB = 4  1-25% SSB = (-1)  26-50% SSB = (-2)  51-75% SSB = (-3)  76-100% SSB = (-4) | Range -4 to 4 | Range -12 to 12 |
|  | 0 CFC = 4  1-3 CFC = (-1)  4-6 CFC = (-2)  7-9 CFC = (-3)  ≥10 CFC = (-4) | Range -4 to 4 | 0% CFC = 4  1-25% CFC = (-1)  26-50% CFC = (-2)  51-75% CFC = (-3)  76-100% CFC = (-4) | Range -4 to 4 | 0% CFC = 4  1-25% CFC = (-1)  26-50% CFC = (-2)  51-75% CFC = (-3)  76-100% CFC = (-4) | Range -4 to 4 | Range -12 to 12 |
|  | 0 FV = (-4)  1-3 FV = (-3)  4-6 FV = (-2)  7-9 FV = (-1)  ≥10 FV = 4 | Range -4 to 4 | 0% FV = (-4)  1-25% FV = 1  26%-50% FV = 2  51-75% FV = 3  76-100% FV = 4 | Range -4 to 4 | N/A |  |  |
| **CHECKOUT END PLACEMENT SCORE** | | | | | | | **Range -48 to 48** |
| **Checkout Side** | 0 CA = 4  1-3 CA = (-1)  4-6 CA = (-2)  7-9 CA = (-3)  ≥10 CA = (-4) | Range -4 to 4 | 0% CA = 4  1-25% CA = (-1)  26-50% CA = (-2)  51-75% CA = (-3)  76-100% CA = (-4) | Range -4 to 4 | 0% CA = 4  1-25% CA = (-1)  26-50% CA = (-2)  51-75% CA = (-3)  76-100% CA = (-4) | Range -4 to 4 | Range -12 to 12 |
|  | 0 CH = 4  1-3 CH = (-1)  4-6 CH = (-2)  7-9 CH = (-3)  ≥10 CH = (-4) | Range -4 to 4 | 0% CH = 4  1-25% CH = (-1)  26-50% CH = (-2)  51-75% CH = (-3)  76-100% CH = (-4) | Range -4 to 4 | 0% CH = 4  1-25% CH = (-1)  26-50% CH = (-2)  51-75% CH = (-3)  76-100% CH = (-4) | Range -4 to 4 | Range -12 to 12 |
|  | 0 SSB = 4  1-3 SSB = (-1)  4-6 SSB = (-2)  7-9 SSB = (-3)  ≥10 SSB = (-4) | Range -4 to 4 | 0% SSB = 4  1-25% SSB = (-1)  26-50% SSB = (-2)  51-75% SSB = (-3)  76-100% SSB = (-4) | Range -4 to 4 | 0% SSB = 4  1-25% SSB = (-1)  26-50% SSB = (-2)  51-75% SSB = (-3)  76-100% SSB = (-4) | Range -4 to 4 | Range -12 to 12 |
|  | 0 CFC = 4  1-3 CFC = (-1)  4-6 CFC = (-2)  7-9 CFC = (-3)  ≥10 CFC = (-4) | Range -4 to 4 | 0% CFC = 4  1-25% CFC = (-1)  26-50% CFC = (-2)  51-75% CFC = (-3)  76-100% CFC = (-4) | Range -4 to 4 | 0% CFC = 4  1-25% CFC = (-1)  26-50% CFC = (-2)  51-75% CFC = (-3)  76-100% CFC = (-4) | Range -4 to 4 | Range -12 to 12 |
|  | 0 FV = (-4)  1-3 FV = (-3)  4-6 FV = (-2)  7-9 FV = (-1)  ≥10 FV = 4 | Range -4 to 4 | 0% FV = (-4)  1-25% FV = 1  26%-50% FV = 2  51-75% FV = 3  76-100% FV = 4 | Range -4 to 4 | N/A | Range -4 to 4 | Range -12 to 12 |
| **CHECKOUT SIDE PLACEMENT SCORE** | | | | | | | **Range -48 to 48** |
| **TOTAL PLACEMENT SCORE** | | | | | | | **Range -288 to 288** |
| *If product not available, do not score for variety or healthy alternative – use 0 for “N/A” | | | | | | | |

| **2. PROMOTIONAL SCORING** | | | | | | | |
| --- | --- | --- | --- | --- | --- | --- | --- |
| Location | Size | Points or N/A* | Theme | Points or N/A* | Display | Points or N/A* | Row Total |
| **Entrance** | 0% CA = 4  1-25% CA = (-1)  26-50% CA = (-2)  51-75% CA = (-3)  76-100% CA = (-4) | Range -4 to 4 | 0% CA = 4  1-25% CA = (-1)  26-50% CA = (-2)  51-75% CA = (-3)  76-100% CA = (-4) | Range -4 to 4 | 0% CA = 4  1-25% CA = (-1)  26-50% CA = (-2)  51-75% CA = (-3)  76-100% CA = (-4) | Range -4 to 4 | Range -12 to 12 |
|  | 0% CH = 4  1-25% CH = (-1)  26-50% CH = (-2)  51-75% CH = (-3)  76-100% CH = (-4) | Range -4 to 4 | 0% CH = 4  1-25% CH = (-1)  26-50% CH = (-2)  51-75% CH = (-3)  76-100% CH = (-4) | Range -4 to 4 | 0% CH = 4  1-25% CH = (-1)  26-50% CH = (-2)  51-75% CH = (-3)  76-100% CH = (-4) | Range -4 to 4 | Range -12 to 12 |
|  | 0% SSB = 4  1-25% SSB = (-1)  26-50% SSB = (-2)  51-75% SSB = (-3)  76-100% SSB = (-4) | Range -4 to 4 | 0% SSB = 4  1-25% SSB = (-1)  26-50% SSB = (-2)  51-75% SSB = (-3)  76-100% SSB = (-4) | Range -4 to 4 | 0% SSB = 4  1-25% SSB = (-1)  26-50% SSB = (-2)  51-75% SSB = (-3)  76-100% SSB = (-4) | Range -4 to 4 | Range -12 to 12 |
|  | 0% CFC = 4  1-25% CFC = (-1)  26-50% CFC = (-2)  51-75% CFC = (-3)  76-100% CFC = (-4) | Range -4 to 4 | 0% CFC = 4  1-25% CFC = (-1)  26-50% CFC = (-2)  51-75% CFC = (-3)  76-100% CFC = (-4) | Range -4 to 4 | 0% CFC = 4  1-25% CFC = (-1)  26-50% CFC = (-2)  51-75% CFC = (-3)  76-100% CFC = (-4) | Range -4 to 4 | Range -12 to 12 |
|  | 0% FV = (-4)  1-25% FV = 1  26%-50% FV = 2  51-75% FV = 3  76-100% FV = 4 | Range -4 to 4 | 0% FV = (-4)  1-25% FV = 1  26%-50% FV = 2  51-75% FV = 3  76-100% FV = 4 | Range -4 to 4 | 0% FV = (-4)  1-25% FV = 1  26%-50% FV = 2  51-75% FV = 3  76-100% FV = 4 | Range -4 to 4 | Range -12 to 12 |
| **ENTRANCE PROMOTIONAL SCORE** | | | | | | | **-60 to 60** |
| **End Cap A** | 0% CA = 4  1-25% CA = (-1)  26-50% CA = (-2)  51-75% CA = (-3)  76-100% CA = (-4) | Range -4 to 4 | 0% CA = 4  1-25% CA = (-1)  26-50% CA = (-2)  51-75% CA = (-3)  76-100% CA = (-4) | Range -4 to 4 | 0% CA = 4  1-25% CA = (-1)  26-50% CA = (-2)  51-75% CA = (-3)  76-100% CA = (-4) | Range -4 to 4 | Range -12 to 12 |
|  | 0% CH = 4  1-25% CH = (-1)  26-50% CH = (-2)  51-75% CH = (-3)  76-100% CH = (-4) | Range -4 to 4 | 0% CH = 4  1-25% CH = (-1)  26-50% CH = (-2)  51-75% CH = (-3)  76-100% CH = (-4) | Range -4 to 4 | 0% CH = 4  1-25% CH = (-1)  26-50% CH = (-2)  51-75% CH = (-3)  76-100% CH = (-4) | Range -4 to 4 | Range -12 to 12 |
|  | 0% SSB = 4  1-25% SSB = (-1)  26-50% SSB = (-2)  51-75% SSB = (-3)  76-100% SSB = (-4) | Range -4 to 4 | 0% SSB = 4  1-25% SSB = (-1)  26-50% SSB = (-2)  51-75% SSB = (-3)  76-100% SSB = (-4) | Range -4 to 4 | 0% SSB = 4  1-25% SSB = (-1)  26-50% SSB = (-2)  51-75% SSB = (-3)  76-100% SSB = (-4) | Range -4 to 4 | Range -12 to 12 |
|  | 0% CFC = 4  1-25% CFC = (-1)  26-50% CFC = (-2)  51-75% CFC = (-3)  76-100% CFC = (-4) | Range -4 to 4 | 0% CFC = 4  1-25% CFC = (-1)  26-50% CFC = (-2)  51-75% CFC = (-3)  76-100% CFC = (-4) | Range -4 to 4 | 0% CFC = 4  1-25% CFC = (-1)  26-50% CFC = (-2)  51-75% CFC = (-3)  76-100% CFC = (-4) | Range -4 to 4 | Range -12 to 12 |
|  | 0% FV = (-4)  1-25% FV = 1  26%-50% FV = 2  51-75% FV = 3  76-100% FV = 4 | Range -4 to 4 | 0% FV = (-4)  1-25% FV = 1  26%-50% FV = 2  51-75% FV = 3  76-100% FV = 4 | Range -4 to 4 | 0% FV = (-4)  1-25% FV = 1  26%-50% FV = 2  51-75% FV = 3  76-100% FV = 4 | Range -4 to 4 | Range -12 to 12 |
| **END CAP A PROMOTIONAL SCORE** | | | | | | | **-60 to 60** |
| **End Cap B** | 0% CA = 4  1-25% CA = (-1)  26-50% CA = (-2)  51-75% CA = (-3)  76-100% CA = (-4) | Range -4 to 4 | 0% CA = 4  1-25% CA = (-1)  26-50% CA = (-2)  51-75% CA = (-3)  76-100% CA = (-4) | Range -4 to 4 | 0% CA = 4  1-25% CA = (-1)  26-50% CA = (-2)  51-75% CA = (-3)  76-100% CA = (-4) | Range -4 to 4 | Range -12 to 12 |
|  | 0% CH = 4  1-25% CH = (-1)  26-50% CH = (-2)  51-75% CH = (-3)  76-100% CH = (-4) | Range -4 to 4 | 0% CH = 4  1-25% CH = (-1)  26-50% CH = (-2)  51-75% CH = (-3)  76-100% CH = (-4) | Range -4 to 4 | 0% CH = 4  1-25% CH = (-1)  26-50% CH = (-2)  51-75% CH = (-3)  76-100% CH = (-4) | Range -4 to 4 | Range -12 to 12 |
|  | 0% SSB = 4  1-25% SSB = (-1)  26-50% SSB = (-2)  51-75% SSB = (-3)  76-100% SSB = (-4) | Range -4 to 4 | 0% SSB = 4  1-25% SSB = (-1)  26-50% SSB = (-2)  51-75% SSB = (-3)  76-100% SSB = (-4) | Range -4 to 4 | 0% SSB = 4  1-25% SSB = (-1)  26-50% SSB = (-2)  51-75% SSB = (-3)  76-100% SSB = (-4) | Range -4 to 4 | Range -12 to 12 |
|  | 0% CFC = 4  1-25% CFC = (-1)  26-50% CFC = (-2)  51-75% CFC = (-3)  76-100% CFC = (-4) | Range -4 to 4 | 0% CFC = 4  1-25% CFC = (-1)  26-50% CFC = (-2)  51-75% CFC = (-3)  76-100% CFC = (-4) | Range -4 to 4 | 0% CFC = 4  1-25% CFC = (-1)  26-50% CFC = (-2)  51-75% CFC = (-3)  76-100% CFC = (-4) | Range -4 to 4 | Range -12 to 12 |
|  | 0% FV = (-4)  1-25% FV = 1  26%-50% FV = 2  51-75% FV = 3  76-100% FV = 4 | Range -4 to 4 | 0% FV = (-4)  1-25% FV = 1  26%-50% FV = 2  51-75% FV = 3  76-100% FV = 4 | Range -4 to 4 | 0% FV = (-4)  1-25% FV = 1  26%-50% FV = 2  51-75% FV = 3  76-100% FV = 4 | Range -4 to 4 | Range -12 to 12 |
| **END CAP B PROMOTIONAL SCORE** | | | | | | | **-60 to 60** |
| **Island** | 0% CA = 4  1-25% CA = (-1)  26-50% CA = (-2)  51-75% CA = (-3)  76-100% CA = (-4) | Range -4 to 4 | 0% CA = 4  1-25% CA = (-1)  26-50% CA = (-2)  51-75% CA = (-3)  76-100% CA = (-4) | Range -4 to 4 | 0% CA = 4  1-25% CA = (-1)  26-50% CA = (-2)  51-75% CA = (-3)  76-100% CA = (-4) | Range -4 to 4 | Range -12 to 12 |
|  | 0% CH = 4  1-25% CH = (-1)  26-50% CH = (-2)  51-75% CH = (-3)  76-100% CH = (-4) | Range -4 to 4 | 0% CH = 4  1-25% CH = (-1)  26-50% CH = (-2)  51-75% CH = (-3)  76-100% CH = (-4) | Range -4 to 4 | 0% CH = 4  1-25% CH = (-1)  26-50% CH = (-2)  51-75% CH = (-3)  76-100% CH = (-4) | Range -4 to 4 | Range -12 to 12 |
|  | 0% SSB = 4  1-25% SSB = (-1)  26-50% SSB = (-2)  51-75% SSB = (-3)  76-100% SSB = (-4) | Range -4 to 4 | 0% SSB = 4  1-25% SSB = (-1)  26-50% SSB = (-2)  51-75% SSB = (-3)  76-100% SSB = (-4) | Range -4 to 4 | 0% SSB = 4  1-25% SSB = (-1)  26-50% SSB = (-2)  51-75% SSB = (-3)  76-100% SSB = (-4) | Range -4 to 4 | Range -12 to 12 |
|  | 0% CFC = 4  1-25% CFC = (-1)  26-50% CFC = (-2)  51-75% CFC = (-3)  76-100% CFC = (-4) | Range -4 to 4 | 0% CFC = 4  1-25% CFC = (-1)  26-50% CFC = (-2)  51-75% CFC = (-3)  76-100% CFC = (-4) | Range -4 to 4 | 0% CFC = 4  1-25% CFC = (-1)  26-50% CFC = (-2)  51-75% CFC = (-3)  76-100% CFC = (-4) | Range -4 to 4 | Range -12 to 12 |
|  | 0% FV = (-4)  1-25% FV = 1  26%-50% FV = 2  51-75% FV = 3  76-100% FV = 4 | Range -4 to 4 | 0% FV = (-4)  1-25% FV = 1  26%-50% FV = 2  51-75% FV = 3  76-100% FV = 4 | Range -4 to 4 | 0% FV = (-4)  1-25% FV = 1  26%-50% FV = 2  51-75% FV = 3  76-100% FV = 4 | Range -4 to 4 | Range -12 to 12 |
| **ISLAND PROMOTIONAL SCORE** | | | | | | | **-60 to 60** |
| **Checkout End** | 0% CA = 4  1-25% CA = (-1)  26-50% CA = (-2)  51-75% CA = (-3)  76-100% CA = (-4) | Range -4 to 4 | 0% CA = 4  1-25% CA = (-1)  26-50% CA = (-2)  51-75% CA = (-3)  76-100% CA = (-4) | Range -4 to 4 | 0% CA = 4  1-25% CA = (-1)  26-50% CA = (-2)  51-75% CA = (-3)  76-100% CA = (-4) | Range -4 to 4 | Range -12 to 12 |
|  | 0% CH = 4  1-25% CH = (-1)  26-50% CH = (-2)  51-75% CH = (-3)  76-100% CH = (-4) | Range -4 to 4 | 0% CH = 4  1-25% CH = (-1)  26-50% CH = (-2)  51-75% CH = (-3)  76-100% CH = (-4) | Range -4 to 4 | 0% CH = 4  1-25% CH = (-1)  26-50% CH = (-2)  51-75% CH = (-3)  76-100% CH = (-4) | Range -4 to 4 | Range -12 to 12 |
|  | 0% SSB = 4  1-25% SSB = (-1)  26-50% SSB = (-2)  51-75% SSB = (-3)  76-100% SSB = (-4) | Range -4 to 4 | 0% SSB = 4  1-25% SSB = (-1)  26-50% SSB = (-2)  51-75% SSB = (-3)  76-100% SSB = (-4) | Range -4 to 4 | 0% SSB = 4  1-25% SSB = (-1)  26-50% SSB = (-2)  51-75% SSB = (-3)  76-100% SSB = (-4) | Range -4 to 4 | Range -12 to 12 |
|  | 0% CFC = 4  1-25% CFC = (-1)  26-50% CFC = (-2)  51-75% CFC = (-3)  76-100% CFC = (-4) | Range -4 to 4 | 0% CFC = 4  1-25% CFC = (-1)  26-50% CFC = (-2)  51-75% CFC = (-3)  76-100% CFC = (-4) | Range -4 to 4 | 0% CFC = 4  1-25% CFC = (-1)  26-50% CFC = (-2)  51-75% CFC = (-3)  76-100% CFC = (-4) | Range -4 to 4 | Range -12 to 12 |
|  | 0% FV = (-4)  1-25% FV = 1  26%-50% FV = 2  51-75% FV = 3  76-100% FV = 4 | Range -4 to 4 | 0% FV = (-4)  1-25% FV = 1  26%-50% FV = 2  51-75% FV = 3  76-100% FV = 4 | Range -4 to 4 | 0% FV = (-4)  1-25% FV = 1  26%-50% FV = 2  51-75% FV = 3  76-100% FV = 4 | Range -4 to 4 | Range -12 to 12 |
| **CHECKOUT END PROMOTIONAL SCORE** | | | | | | | **-60 to 60** |
| **Checkout Side** | 0% CA = 4  1-25% CA = (-1)  26-50% CA = (-2)  51-75% CA = (-3)  76-100% CA = (-4) | Range -4 to 4 | 0% CA = 4  1-25% CA = (-1)  26-50% CA = (-2)  51-75% CA = (-3)  76-100% CA = (-4) | Range -4 to 4 | 0% CA = 4  1-25% CA = (-1)  26-50% CA = (-2)  51-75% CA = (-3)  76-100% CA = (-4) | Range -4 to 4 | Range -12 to 12 |
|  | 0% CH = 4  1-25% CH = (-1)  26-50% CH = (-2)  51-75% CH = (-3)  76-100% CH = (-4) | Range -4 to 4 | 0% CH = 4  1-25% CH = (-1)  26-50% CH = (-2)  51-75% CH = (-3)  76-100% CH = (-4) | Range -4 to 4 | 0% CH = 4  1-25% CH = (-1)  26-50% CH = (-2)  51-75% CH = (-3)  76-100% CH = (-4) | Range -4 to 4 | Range -12 to 12 |
|  | 0% SSB = 4  1-25% SSB = (-1)  26-50% SSB = (-2)  51-75% SSB = (-3)  76-100% SSB = (-4) | Range -4 to 4 | 0% SSB = 4  1-25% SSB = (-1)  26-50% SSB = (-2)  51-75% SSB = (-3)  76-100% SSB = (-4) | Range -4 to 4 | 0% SSB = 4  1-25% SSB = (-1)  26-50% SSB = (-2)  51-75% SSB = (-3)  76-100% SSB = (-4) | Range -4 to 4 | Range -12 to 12 |
|  | 0% CFC = 4  1-25% CFC = (-1)  26-50% CFC = (-2)  51-75% CFC = (-3)  76-100% CFC = (-4) | Range -4 to 4 | 0% CFC = 4  1-25% CFC = (-1)  26-50% CFC = (-2)  51-75% CFC = (-3)  76-100% CFC = (-4) | Range -4 to 4 | 0% CFC = 4  1-25% CFC = (-1)  26-50% CFC = (-2)  51-75% CFC = (-3)  76-100% CFC = (-4) | Range -4 to 4 | Range -12 to 12 |
|  | 0% FV = (-4)  1-25% FV = 1  26%-50% FV = 2  51-75% FV = 3  76-100% FV = 4 | Range -4 to 4 | 0% FV = (-4)  1-25% FV = 1  26%-50% FV = 2  51-75% FV = 3  76-100% FV = 4 | Range -4 to 4 | 0% FV = (-4)  1-25% FV = 1  26%-50% FV = 2  51-75% FV = 3  76-100% FV = 4 | Range -4 to 4 | Range -12 to 12 |
| **CHECKOUT SIDE PROMOTIONAL SCORE** | | | | | | | **-60 to 60** |
| **TOTAL PROMOTIONAL SCORE** | | | | | | | **-360 to 360** |
| *If product not available, do not score for size, theme, or display – use 0 for “N/A” | | | | | | | |

| **3. CHILD MARKETING SCORING** | | | | | |
| --- | --- | --- | --- | --- | --- |
| Location | Child Height | Points or N/A* | Child Focused | Points or N/A* | Row Total |
| **Entrance** | 0% CA = 4  1-25% CA = (-1)  26-50% CA = (-2)  51-75% CA = (-3)  76-100% CA = (-4) | Range -4 to 4 | 0% CA = 4  1-25% CA = (-1)  26-50% CA = (-2)  51-75% CA = (-3)  76-100% CA = (-4) | Range -4 to 4 | -8 to 8 |
|  | 0% CH = 4  1-25% CH = (-1)  26-50% CH = (-2)  51-75% CH = (-3)  76-100% CH = (-4) | Range -4 to 4 | 0% CH = 4  1-25% CH = (-1)  26-50% CH = (-2)  51-75% CH = (-3)  76-100% CH = (-4) | Range -4 to 4 | -8 to 8 |
|  | 0% SSB = 4  1-25% SSB = (-1)  26-50% SSB = (-2)  51-75% SSB = (-3)  76-100% SSB = (-4) | Range -4 to 4 | 0% SSB = 4  1-25% SSB = (-1)  26-50% SSB = (-2)  51-75% SSB = (-3)  76-100% SSB = (-4) | Range -4 to 4 | -8 to 8 |
|  | 0% CFC = 4  1-25% CFC = (-1)  26-50% CFC = (-2)  51-75% CFC = (-3)  76-100% CFC = (-4) | Range -4 to 4 | 0% CFC = 4  1-25% CFC = (-1)  26-50% CFC = (-2)  51-75% CFC = (-3)  76-100% CFC = (-4) | Range -4 to 4 | -8 to 8 |
|  | 0% FV = (-4)  1-25% FV = 1  26%-50% FV = 2  51-75% FV = 3  76-100% FV = 4 | Range -4 to 4 | 0% FV = (-4)  1-25% FV = 1  26%-50% FV = 2  51-75% FV = 3  76-100% FV = 4 | Range -4 to 4 | -8 to 8 |
| **ENTRANCE CHILD MARKETING SCORE** | | | | | **-40 to 40** |
| **End Cap A** | 0% CA = 4  1-25% CA = (-1)  26-50% CA = (-2)  51-75% CA = (-3)  76-100% CA = (-4) | Range -4 to 4 | 0% CA = 4  1-25% CA = (-1)  26-50% CA = (-2)  51-75% CA = (-3)  76-100% CA = (-4) |  | -8 to 8 |
|  | 0% CH = 4  1-25% CH = (-1)  26-50% CH = (-2)  51-75% CH = (-3)  76-100% CH = (-4) | Range -4 to 4 | 0% CH = 4  1-25% CH = (-1)  26-50% CH = (-2)  51-75% CH = (-3)  76-100% CH = (-4) |  | -8 to 8 |
|  | 0% SSB = 4  1-25% SSB = (-1)  26-50% SSB = (-2)  51-75% SSB = (-3)  76-100% SSB = (-4) | Range -4 to 4 | 0% SSB = 4  1-25% SSB = (-1)  26-50% SSB = (-2)  51-75% SSB = (-3)  76-100% SSB = (-4) |  | -8 to 8 |
|  | 0% CFC = 4  1-25% CFC = (-1)  26-50% CFC = (-2)  51-75% CFC = (-3)  76-100% CFC = (-4) | Range -4 to 4 | 0% CFC = 4  1-25% CFC = (-1)  26-50% CFC = (-2)  51-75% CFC = (-3)  76-100% CFC = (-4) |  | -8 to 8 |
|  | 0% FV = (-4)  1-25% FV = 1  26%-50% FV = 2  51-75% FV = 3  76-100% FV = 4 | Range -4 to 4 | 0% FV = (-4)  1-25% FV = 1  26%-50% FV = 2  51-75% FV = 3  76-100% FV = 4 |  | -8 to 8 |
| **END CAP A CHILD MARKETING SCORE** | | | | | **-40 to 40** |
| **End Cap B** | 0% CA = 4  1-25% CA = (-1)  26-50% CA = (-2)  51-75% CA = (-3)  76-100% CA = (-4) | Range -4 to 4 | 0% CA = 4  1-25% CA = (-1)  26-50% CA = (-2)  51-75% CA = (-3)  76-100% CA = (-4) | Range -4 to 4 | -8 to 8 |
|  | 0% CH = 4  1-25% CH = (-1)  26-50% CH = (-2)  51-75% CH = (-3)  76-100% CH = (-4) | Range -4 to 4 | 0% CH = 4  1-25% CH = (-1)  26-50% CH = (-2)  51-75% CH = (-3)  76-100% CH = (-4) | Range -4 to 4 | -8 to 8 |
|  | 0% SSB = 4  1-25% SSB = (-1)  26-50% SSB = (-2)  51-75% SSB = (-3)  76-100% SSB = (-4) | Range -4 to 4 | 0% SSB = 4  1-25% SSB = (-1)  26-50% SSB = (-2)  51-75% SSB = (-3)  76-100% SSB = (-4) | Range -4 to 4 | -8 to 8 |
|  | 0% CFC = 4  1-25% CFC = (-1)  26-50% CFC = (-2)  51-75% CFC = (-3)  76-100% CFC = (-4) | Range -4 to 4 | 0% CFC = 4  1-25% CFC = (-1)  26-50% CFC = (-2)  51-75% CFC = (-3)  76-100% CFC = (-4) | Range -4 to 4 | -8 to 8 |
|  | 0% FV = (-4)  1-25% FV = 1  26%-50% FV = 2  51-75% FV = 3  76-100% FV = 4 | Range -4 to 4 | 0% FV = (-4)  1-25% FV = 1  26%-50% FV = 2  51-75% FV = 3  76-100% FV = 4 | Range -4 to 4 | -8 to 8 |
| **END CAP B CHILD MARKETING SCORE** | | | | | **-40 to 40** |
| **Island** | 0% CA = 4  1-25% CA = (-1)  26-50% CA = (-2)  51-75% CA = (-3)  76-100% CA = (-4) | Range -4 to 4 | 0% CA = 4  1-25% CA = (-1)  26-50% CA = (-2)  51-75% CA = (-3)  76-100% CA = (-4) | Range -4 to 4 | -8 to 8 |
|  | 0% CH = 4  1-25% CH = (-1)  26-50% CH = (-2)  51-75% CH = (-3)  76-100% CH = (-4) | Range -4 to 4 | 0% CH = 4  1-25% CH = (-1)  26-50% CH = (-2)  51-75% CH = (-3)  76-100% CH = (-4) | Range -4 to 4 | -8 to 8 |
|  | 0% SSB = 4  1-25% SSB = (-1)  26-50% SSB = (-2)  51-75% SSB = (-3)  76-100% SSB = (-4) | Range -4 to 4 | 0% SSB = 4  1-25% SSB = (-1)  26-50% SSB = (-2)  51-75% SSB = (-3)  76-100% SSB = (-4) | Range -4 to 4 | -8 to 8 |
|  | 0% CFC = 4  1-25% CFC = (-1)  26-50% CFC = (-2)  51-75% CFC = (-3)  76-100% CFC = (-4) | Range -4 to 4 | 0% CFC = 4  1-25% CFC = (-1)  26-50% CFC = (-2)  51-75% CFC = (-3)  76-100% CFC = (-4) | Range -4 to 4 | -8 to 8 |
|  | 0% FV = (-4)  1-25% FV = 1  26%-50% FV = 2  51-75% FV = 3  76-100% FV = 4 | Range -4 to 4 | 0% FV = (-4)  1-25% FV = 1  26%-50% FV = 2  51-75% FV = 3  76-100% FV = 4 | Range -4 to 4 | -8 to 8 |
| **ISLAND CHILD MARKETING SCORE** | | | | | **-40 to 40** |
| **Checkout End** | 0% CA = 4  1-25% CA = (-1)  26-50% CA = (-2)  51-75% CA = (-3)  76-100% CA = (-4) | Range -4 to 4 | 0% CA = 4  1-25% CA = (-1)  26-50% CA = (-2)  51-75% CA = (-3)  76-100% CA = (-4) | Range -4 to 4 | -8 to 8 |
|  | 0% CH = 4  1-25% CH = (-1)  26-50% CH = (-2)  51-75% CH = (-3)  76-100% CH = (-4) | Range -4 to 4 | 0% CH = 4  1-25% CH = (-1)  26-50% CH = (-2)  51-75% CH = (-3)  76-100% CH = (-4) | Range -4 to 4 | -8 to 8 |
|  | 0% SSB = 4  1-25% SSB = (-1)  26-50% SSB = (-2)  51-75% SSB = (-3)  76-100% SSB = (-4) | Range -4 to 4 | 0% SSB = 4  1-25% SSB = (-1)  26-50% SSB = (-2)  51-75% SSB = (-3)  76-100% SSB = (-4) | Range -4 to 4 | -8 to 8 |
|  | 0% CFC = 4  1-25% CFC = (-1)  26-50% CFC = (-2)  51-75% CFC = (-3)  76-100% CFC = (-4) | Range -4 to 4 | 0% CFC = 4  1-25% CFC = (-1)  26-50% CFC = (-2)  51-75% CFC = (-3)  76-100% CFC = (-4) | Range -4 to 4 | -8 to 8 |
|  | 0% FV = (-4)  1-25% FV = 1  26%-50% FV = 2  51-75% FV = 3  76-100% FV = 4 | Range -4 to 4 | 0% FV = (-4)  1-25% FV = 1  26%-50% FV = 2  51-75% FV = 3  76-100% FV = 4 | Range -4 to 4 | -8 to 8 |
| **CHECKOUT END CHILD MARKETING SCORE** | | | | | **-40 to 40** |
| **Checkout Side** | 0% CA = 4  1-25% CA = (-1)  26-50% CA = (-2)  51-75% CA = (-3)  76-100% CA = (-4) | Range -4 to 4 | 0% CA = 4  1-25% CA = (-1)  26-50% CA = (-2)  51-75% CA = (-3)  76-100% CA = (-4) | Range -4 to 4 | -8 to 8 |
|  | 0% CH = 4  1-25% CH = (-1)  26-50% CH = (-2)  51-75% CH = (-3)  76-100% CH = (-4) | Range -4 to 4 | 0% CH = 4  1-25% CH = (-1)  26-50% CH = (-2)  51-75% CH = (-3)  76-100% CH = (-4) | Range -4 to 4 | -8 to 8 |
|  | 0% SSB = 4  1-25% SSB = (-1)  26-50% SSB = (-2)  51-75% SSB = (-3)  76-100% SSB = (-4) | Range -4 to 4 | 0% SSB = 4  1-25% SSB = (-1)  26-50% SSB = (-2)  51-75% SSB = (-3)  76-100% SSB = (-4) | Range -4 to 4 | -8 to 8 |
|  | 0% CFC = 4  1-25% CFC = (-1)  26-50% CFC = (-2)  51-75% CFC = (-3)  76-100% CFC = (-4) | Range -4 to 4 | 0% CFC = 4  1-25% CFC = (-1)  26-50% CFC = (-2)  51-75% CFC = (-3)  76-100% CFC = (-4) | Range -4 to 4 | -8 to 8 |
|  | 0% FV = (-4)  1-25% FV = 1  26%-50% FV = 2  51-75% FV = 3  76-100% FV = 4 | Range -4 to 4 | 0% FV = (-4)  1-25% FV = 1  26%-50% FV = 2  51-75% FV = 3  76-100% FV = 4 | Range -4 to 4 | -8 to 8 |
| **CHECKOUT SIDE CHILD MARKETING SCORE** | | | | | **-40 to 40** |
| **TOTAL CHILD MARKETING SCORE** | | | | | **-240 to 240** |
| *If product not available, do not score for child height or child focus – use 0 for “N/A” | | | | | |
